# Separating “good” from “bad” faecal dysbiosis – evidence from two cross-sectional studies

**DOI:** 10.1186/s40608-018-0207-3

**Published:** 2018-12-03

**Authors:** Per G. Farup, Martin Aasbrenn, Jørgen Valeur

**Affiliations:** 1 Department of Research, Innlandet Hospital Trust, N-2381 Brumunddal, Norway; 2 Unit for Applied Clinical Research, Department of Clinical and Molecular Medicine, Faculty of Medicine and Health Sciences, Norwegian University of Science and Technology, N-7491 Trondheim, Norway; 3 Department of Surgery, Innlandet Hospital Trust, N-2819 Gjøvik, Norway; 4 Novo Nordisk Foundation Center for Basic Metabolic Research, Section of Metabolic Genetics, Faculty of Health and Medical Sciences, University of Copenhagen, DK-2200 Copenhagen, Denmark; 5 Unger-Vetlesen Institute, Lovisenberg Diaconal Hospital, N-0440 Oslo, Norway

**Keywords:** Dysbiosis, Irritable bowel syndrome, Metformin, Microbiota, Non-nutritive sweeteners; obesity

## Abstract

**Background:**

Faecal dysbiosis associated with the use of metformin has been conceived as a favourable (“good”) dysbiosis and that with intake of non-nutritive sweeteners (NNS) as unfavourable (“bad”). The study aimed to construct an alternative dysbiosis index (ADI) for the separation of the dysbioses into “good” and “bad”, and to validate the ADI.

**Methods:**

Subjects with morbid obesity were included. Use of NNS and drugs were noted, IBS was classified according to the Rome III criteria and the severity measured with the Irritable bowel severity scoring system (IBSSS). Faecal dysbiosis was tested with GA-Map ™ Dysbiosis test (Genetic Analysis AS, Oslo, Norway). The result was given as Dysbiosis Index (DI) scores 1–5, score > 2 indicates dysbiosis. An ADI was constructed and validated in subjects with IBS at another hospital.

**Results:**

Seventy-six women and 14 men aged 44.7 years (SD 8.6) with BMI 41.8 kg/m^2^ (SD 3.6) were included. Dysbiosis was associated with the use of NNS and metformin, but not with IBS or IBSSS. An ADI based on differences in 7 bacteria was positively and negatively associated with the “good” metformin dysbiosis and the “bad” NNS dysbiosis respectively. The ADI was also negatively associated with IBSSS (a “bad” dysbiosis). The negative associations between ADI and IBS and IBSS were confirmed in the validation group.

**Conclusions:**

The new ADI, but not the DI, allowed separation of the “good” and “bad” faecal dysbiosis. Rather than merely reporting dysbiosis and degrees of dysbiosis, future diagnostic tests should distinguish between types of dysbiosis.

## Background

The gut microbiota interferes with the mucosal immune system, the cytokine secretion, the intestinal permeability, the secretion of mucus, antimicrobial peptides and IgA, and the production of metabolites and other unknown factors. Gut dysbiosis, defined as an imbalance or deviation from the normal composition of the microbiota, might be either beneficial (good) due to improved immune system, increased anti/pro inflammatory cytokine ratio etc., or deleterious (bad). Dysbiosis has been associated with and mentioned as a causal factor for obesity in humans [1, 2]. Dysbiosis has also been suggested as a causal factor for insulin resistance, glucose intolerance and type 2 diabetes, which are common comorbidities in in subjects with morbid obesity [1, 3]. These types of dysbiosis are “bad”.

Both the diet and drugs influence the faecal microbiota [4–7]. Metformin has anti-hyperglycemic and weight-reducing effects, which are beneficial in subjects with obesity [8–10]. The effects depend in part on the altering of the gut microbiome [11–13]. The metformin-induced dysbiosis, therefore, contributes to the therapeutic effects and is referred to as “good” dysbiosis.

To prevent weight gain and facilitate weight reduction, subjects with obesity have a high intake of non-nutritive sweeteners (NNS) [14]. NNS induce glucose intolerance by altering the gut microbiota and has been linked to obesity by the obesity-associated metabolic changes [15–17]. Therefore, the dysbiosis associated with NNS seems to be unfavourable and is henceforth denoted as “bad” dysbiosis.

Irritable bowel syndrome (IBS), a common comorbidity in subjects with morbid obesity, is one of many disorders associated with alterations in the gut microbiota (“bad” dysbiosis) [18–21]. In all, dysbiosis is associated with various disorders. According to the recently proposed “Anna Karenina principle” for animal microbiomes, the microbiome varies more in dysbiotic than in healthy subjects, and such variations might be separated into “good” and “bad” dysbioses [22].

Today’s knowledge about dysbiosis is limited, the diagnostic tests are complicated and expensive, and the clinical utility is questionable. Knowledge of “good” and “bad” dysbioses might have clinical implications, such as normalising or preventing the “bad” dysbioses and preserving the “good” ones.

A simplified test for faecal dysbiosis based upon 54 DNA probes targeting gut bacteria has been marketed in Europe and USA (GA-map™ Dysbiosis Test, manufactured by Genetic Analysis, Oslo, Norway) [23, 24].

In this study, the primary aims were to assess the commercially available dysbiosis test’s ability to detect faecal dysbiosis in subjects with morbid obesity and to detect dysbiosis associated with other variables, primarily metformin, NNS, diabetes, IBS and gastrointestinal symptoms. Based on the hypothesis that the dysbioses associated with the use of metformin and NNS differed [22], the secondary aims were to use the results of the dysbiosis test to explore alternative scoring algorithms to detect differences between the dysbiosis associated with metformin and NNS. The alternative scoring was validated in a new cross-sectional study.

## Methods

### Study design

Exploratory analyses were performed in one cross-sectional study (the test group) and validated in another cross-sectional study (the validation group).

In the test group, the dysbiosis test’s ability to detect dysbiosis related to obesity, diabetes, IBS, the severity of gastrointestinal symptoms and use of NNS and metformin were studied. If dysbiosis was detected, explorative analyses were performed to detect differences between the dysbioses related to metformin (the “good” dysbiosis) and NNS (the “bad” dysbiosis) and to work out an Alternative Dysbiosis Index that distinguished between the “good” and “bad” dysbioses. Some of the results were validated in the validation group.

### Subjects

From December 2012 to September 2014, consecutive subjects aged 18–65 years with morbid obesity (defined as BMI ≥ 40 kg/m^2^ or ≥ 35 kg/m^2^ with obesity-related complications) were included in the test group at Innlandet Hospital Trust, Gjøvik, Norway. At Lovisenberg Diaconal Hospital’s outpatient clinic for gastrointestinal disorders, consecutive subjects above 18 years of age with IBS were from April 2013 to October 2014 included in the validation group. At both centres, a medical history was taken, paper-based questionnaires were filled in by the patients, a physical examination was performed, and blood and faecal samples were collected. Supplementary examinations were performed at the doctors’ discretion. Subjects with serious somatic and psychiatric disorders (if judged as unrelated to obesity in the test group) were excluded because they could confound the evaluation of dysbiosis, and subjects with previous major abdominal surgery including bariatric surgery were excluded to ascertain the diagnosis of IBS. In addition, subjects not delivering faecal samples, subjects with incompletely filled in food frequency questionnaires (FFQ) were excluded from the test group, and subjects using antibiotics the last month or with a ^13^C-D-Xylose breath test indicating malabsorption were excluded from the validation group. At both centres, trained personnel was responsible for the care of the patients and the practical work.

### Variables

Gender, age (years), body weight (kg), height (meter), body mass index (BMI, kg/m^2^), smoking habits (never / previously / daily smokers), and present or previous somatic disorders including hypertension, diabetes, and hypothyroidism (yes / no) were noted. Irritable Bowel Syndrome (IBS) was diagnosed with a validated Norwegian translation of the Rome III criteria, and the degree of gastrointestinal complaints with Irritable Bowel Severity Scoring System (IBSSS) [25]. The use of metformin, statins, and thyroxin was recorded. A range of haematological and biochemical blood tests including vitamins and minerals were analysed.

The dietary intake of micro- and macro nutrients, energy, and NNS were assessed with an FFQ prepared, validated and analyzed by the Department of Nutrition at the University of Oslo, Norway. The analyses were performed with their in-house calculation program (KBS, version 7.3, food database AE-14) based on the official Norwegian food composition table from 2016 [26]. The intake of NNS was calculated pragmatically since the FFQ did not specify the type or amount of NNS in the beverages. One unit of NNS was defined as 100 ml NNS-containing beverage (divided into carbonated and non-carbonated beverage) which was considered equal to the sweetening of sugar-containing beverages with 10% of sugar (10 g/100 ml). Two NNS tablets/teaspoons for use in tea or coffee were judged as equal to 100 ml NNS in beverages. The unit (100 ml beverages or two tablets/teaspoons) could easily be calculated since the subjects reported the intake in litre and glasses, and the unit is easily understood. Intakes of NNS from other sources than beverages and tablets/teaspoons used in beverages were not recorded. Sugar alcohols and naturally-derived sweeteners not defined as NNS were not included. In addition to the associations between dysbiosis and NNS, the associations between dysbiosis and sugar-containing beverages and the absolute and relative amounts of macronutrients were analysed.

The faecal microbiota was analysed with the CE marked GA-map™ Dysbiosis Test (Genetic Analysis AS, Oslo, Norway) [23]. The test has both a US (Patent No. 9243297) and European patent (Patent No. 2652145) for its technology governing the oligonucleotide probe set and methods of microbiota profiling [24]. It uses 54 oligonucleotide probes targeting the 16S rRNA gene at different bacterial taxonomic levels and scores the relative abundance of each bacteria compared to the distribution in a reference population (score −3 to 3). The overall result is given as the Dysbiosis Index (DI) with scores 1 to 5, where values above 2 indicate a microbiota profile that differs from the reference population (i.e. dysbiosis). Exploratory analyses were performed to show differences between metformin and NNS in the relative abundance of one or more of the bacteria measured on the score from −3 to 3. The detected differences were summarised in the ADI.

### Statistics

The results have been reported as mean (SD), median (range), and number (proportion in percentage). Comparisons between groups were analysed with chi-square tests, t-test, Mann-Whitney U-test, Pearson’s and Spearman’s correlation analyses depending on type and distribution of the data. Independent predictors of dysbiosis were assessed with linear regression analyses including gender, BMI and all variables significantly associated with dysbiosis in the univariable analyses followed by stepwise forward regression analyses. The results of the linear regression analyses are given as B-value with 95% confidence interval (CI), *p*-value and partial correlation (pc). The analyses were performed with IBM SPSS Statistics for Windows, Version 25.0. Armonk, NY: IBM Corp. *p*-values < 0.05 were judged as statistically significant.

## Results

### The test group

**Fig. 1:**
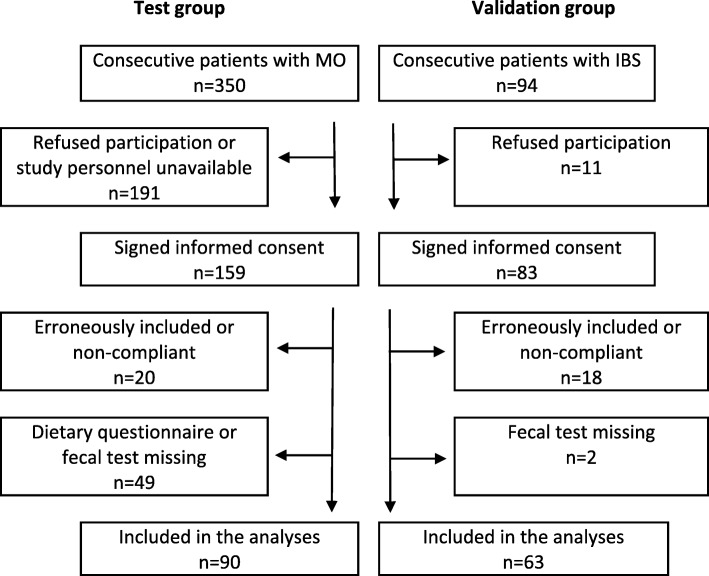
A flowchart of the subjects in the study

**Fig. 2:**
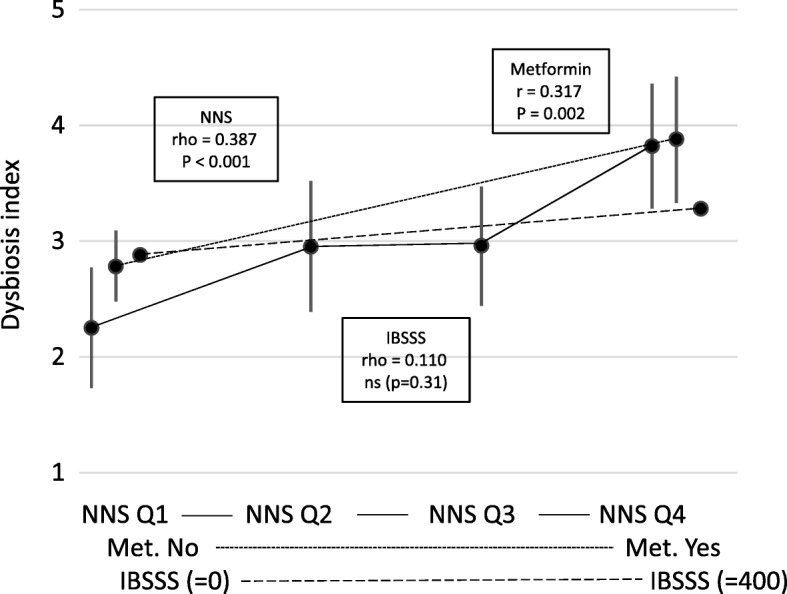
Associations between the Dysbiosis Index and the main variables. NNS Q1, NNS Q2, NNS Q3, NNS Q4: Intake of Non-Nutritive Sweeteners divided into quartiles. Met: Metformin. IBSSS: Irritable Bowel Severity Scoring System. The results for NNS and Met are given as mean with 95% CI. The associations are given as Pearson’s and Spearman's correlation coefficients (r and rho) and significance value (*p*-value)

Out of 350 consecutive subjects visiting the obesity unit, 90 (76 women and 14 men with a mean age of 44.7 years (SD 8.6) and BMI 41.8 kg/m2 (SD 3.6)) were included in the test group. The reasons for the exclusion of 260 subjects are given in Fig. 1. Table 1 gives the participants’ characteristics in detail divided into subjects with and without dysbiosis. Dysbiosis was present in 59 (66%) of the subjects; the mean DI score was 3.0 (SD 1.3). The DI scores 1–5 were present in 16 (18%), 15 (17%), 30 (33%), 13 (14%), and 16 (18%) subjects respectively. The main finding was the associations between dysbiosis and diabetes, metformin and NNS (all *p*-values < 0.01). There were no significant associations with either IBS or IBSSS. Table 1 gives all the associations except for the associations with the relative amounts of the macronutrients since there were no significant associations with these variables. Figure 2 shows the associations between the DI and the use of NNS and metformin and IBSSS.

**Table 1:** The characteristics of the participants in the study and associations with dysbiosis

Participants’ characteristics^a^	Dysbiosis		Dysbiosis	Association with DI ^b^	Association with ADI ^b^
	Yes (no 59)	No (31)	*p*-values		
Gender (female/male)	49 (83%) / 10 (17%)	27 (87%) / 4 (13%)	0.76 ^(1)^	2.9 (1.3) / 3.4 (1.5); *p* = 0.17 ^(2)^	−0.9 (2.8) / −0.5 (2.7); *p* = 0.66 ^(2)^
Age (years)	44.0 (8.6)	46.0 (8.6)	0.31 ^(2)^	*r* = −0.078; *p* = 0.47	*r* = −0.375; *p < 0.001*
Body weight (kg)	120.5 (15.2)	122.1 (18.3)	0.66^(2)^	*r* = −0.064; *p* = 0.55	*r* = 0.009; *p* = 0.93
BMI (kg/m2)	42.1 (3.7)	41.2 (3.2)	0.30 ^(2)^	*r* = 0.022; *p* = 0.84	*r* = −0.186; *p* = 0.08
Smoking (never/previously /daily)	19 (32%) / 31 (53%) / 9 (15%)	17 (55%) / 12 (39%) / 2 (6%)	*0.046* ^(3)^	*r* = 0.195; *p* = 0.07	*r* = −0.230; *p* = *0.030*
Coffee (cups/day)	2.6 (2.3)	3.5 (2.4)	0.08 ^(2)^	rho = −0.240; *p = 0.026*	rho = 0.163; *p* = 0.13
Food intolerance (65)	33/43 (77%)	17/22 (77%)	1.00 ^(1)^	3.0 (1.3) / 3.0 (1.5); *p* = 0.95 ^(2)^	−1.1 (2.5) / −1.7 (3.0); *p* = 0.45 ^(2)^
Diabetes (87)	18/56 (32%)	2/31 (7%)	*0.007* ^(1)^	3.7 (1.1) / 2.8 (1.3); *p* *= 0.005*^(2)^	0.5 (3.3) / −1.1 (2.5); *p* = 0.057 ^(2)^
Hypothyreosis (86)	7/56 (13%)	3/30 (10%)	1.00 ^(1)^	3.5 (1.3) / 2.9 (1.3); *p* = 0.19 ^(2)^	−0.1 (2.8) / −0.8 (2.8); *p* = 0.46 ^(2)^
IBS (88)	17/59 (29%)	8/29 (28%	1.00 ^(1)^	3.1 (1.3) / 3.0 (1.4); *p* = 0.60	−1.6 (2.8) / −0.4 (2.7); *p* = 0.063 ^(2)^
IBSSS (86)	120 (0–389)	99 (0–339)	0.43 ^(4)^	rho = 0.110; *p* = 0.31	rho = −0.304; *p* *= 0.004*
Metformin	15/59 (25%)	1/31 (3%)	*0.009* ^(1)^	3.9 (1.0) / 2.8 (1.3); *p* *= 0.002*^(2)^	1.1 (3.1) / −1.2 (2.5); *p* *= 0.002*^(2)^
Thyroxin (89)	5 / 59 (9%)	2/30 (7%)	1.00 ^(1)^	3.6 (1.3) / 2.9 (1.3); *p* = 0.23 ^(2)^	0.1 (2.9) / −0.9 (2.8); *p* = 0.33 ^(2)^
Statins (88)	8 / 58 (14%)	5 / 13 (17%)	0.76 ^(1)^	2.5 (1.3) / 3.1 (1.3); *p* = 0.19 ^(2)^	−0.3 (2.3) / −0.9 (2.8); *p* = 0.47 ^(2)^
CRP (88)	6 (0–27)	5 (1–28)	0.34 ^(4)^	rho = 0.076; *p* = 0.48	rho = −0.034; *p* = 0.75
Zonulin (ng/ml) (85)	71 (36)	60 (30)	0.14 ^(2)^	*r* = 0.179; *p* = 0.10	*r* = −0.125; *p* = 0.25
Total energy (kJ)	10,387 (3875)	10,777 (4324)	0.66 ^(2)^	*r* = −0.044; *p* = 0.68	*r* = −0.236; *p* *= 0.025*
Water (unit)	44 (21)	42 (19)	0.65 ^(2)^	*r* = 0.030; *p* = 0.78	*r* = −0.251; *p* *= 0.017*
Carb. beverages w/ sugar (unit^c^)	0.0 (0–7.9)	0 (0–2.6)	0.99 ^(4)^	rho = −0.016; *p* = 0.89	rho = −0.220; *p* *= 0.037*
Total NNS (unit^c^)	4.9 (0–43)	1 (0–22)	*0.002* ^(4)^	rho = 0.387; *p < 0.001*	rho = −0.353; *p* =* 0.001*
Carb. beverages w/NNS (unit^c^)	1.1 (0–40)	0 (0–20)	*0.002* ^(4)^	rho = 0.378; *p < 0.001*	rho = −0.268; *p = 0.011*
Non-carb. Beverages w/NNS (unit^c^)	0.4 (0–32)	0 (0–8)	*0.026* ^(4)^	rho = 0.270; *p = 0.010*	rho = −0.240; *p = 0.023*
Coffee/tea w/NNS (unit^c^)	0.0 (0–27)	0 (0–14)	0.73 ^(4)^	rho = −0.047; *p* = 0.66	rho = −0.151; *p* = 0.16
Protein (g/day)	110 (36)	110 (36)	0.94 ^(2)^	rho = −0.053; *p* = 0.62	rho = −0.156; *p* = 0.14
Fat (g/day)	95 (42)	103 (56)	0.45 ^(2)^	rho = −0.048; *p* = 0.66	rho = −0.235; *p = 0.026*
Carbohydrates (g/day)	273 (119)	280 (150)	0.80 ^(2)^	rho = 0.016; *p* = 0.88	rho = −0.200; *p* = 0.058
Starch (g/day)	133 (54)	131 (46)	0.83 ^(2)^	rho = 0.035; *p* = 0.74	rho = −0.263; *p = 0.012*
Sugar (g/day)	25 (1–268)	32 (5–632)	0.99	rho = 0.012; *p* = 0.91	rho = −0.221; *p = 0.037*

*DI* Dysbiosis Index, *ADI* Alternative Dysbiosis Index, *NNS* Non-Nutritive Sweeteners. The results are given as mean (SD); median (range); number (%). ^a^The number of subjects is given in brackets if less than 90. ^b^The order of the results are yes/no.^c^ One unit is 100 ml beverages with NNS or 2 tablets NNS for coffee/tea

^(1)^: Fisher’s exact test; ^(2)^ t-test; ^(3)^ chi-square, linear-by-linear; ^(4)^ Exact Mann-Whitney; rho: Spearman’s rho; r = Pearson Correlation test

Italicized *p*-values are statistically significant

**Fig. 3:**
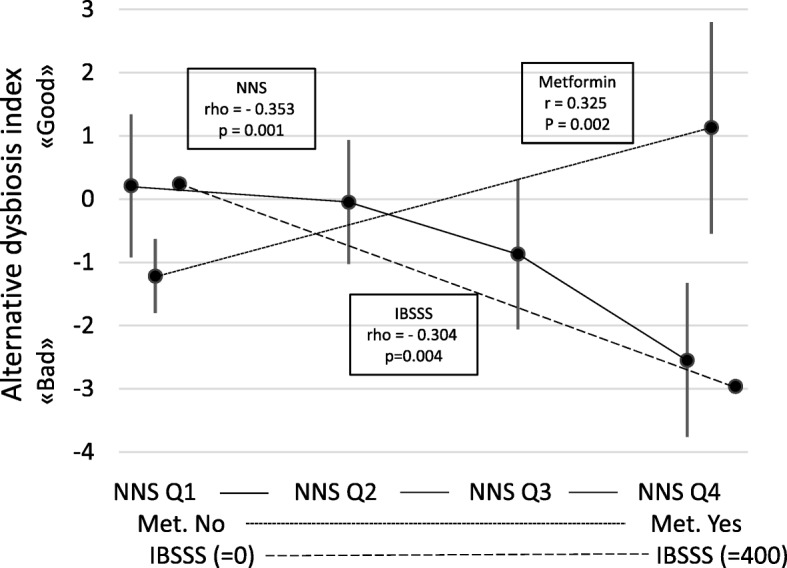
Associations between the Alternative Dysbiosis Index and the main variables. NNS Q1, NNS Q2, NNS Q3, NNS Q4: Intake of Non-Nutritive Sweeteners divided into quartiles. Met: Metformin. IBSSS: Irritable Bowel Severity Scoring System. The results for NNS and Met are given as mean with 95% CI. The associations are given as Pearson’s and Spearman's correlation coefficients (r and rho) and significance value (*p*-value)

Explorative analyses revealed significant differences between the dysbiosis related to NNS and metformin. Compared to NNS, the dysbiosis related to metformin was characterised by a relative abundance of the bacteria Alistipes, Proteobacteria and Shigella spp. & Escherichia spp., and a relative scarcity of *Bacteroides fragilis*, *Ruminococcus gnavus*, Bacteroides spp. & Prevotella spp., and *Dialister invisus*. The signs of the scores for the bacteria with a relative scarcity were changed. Then the scores for the seven bacteria were summed up and adjusted to the Alternative Dysbiosis Index (ADI) with scores from −14 to 14; positive scores were associated with the use of metformin (the “good” dysbiosis) and negative scores with the use of NNS (the “bad” dysbiosis). The mean ADI score was −0.8 (SD 2.8). Table 1 gives all associations between the patients’ characteristics and the ADI. Figure 3 shows the positive association between the ADI and metformin and the negative associations with NNS and IBSSS, which were the main and statistically significant findings.

Multivariable analyses were used to study independent predictors of DI and ADI. These analyses included gender and BMI and all variables with a significant association with either DI or ADI. Diabetes and metformin were highly correlated (*r* = 0.80). Because the associations between metformin and DI and ADI were significantly higher than between diabetes and DI and ADI, diabetes was excluded from the analyses. Separate multivariable analyses (not shown) of the associations between DI and ADI on one side and total energy intake and the absolute and relative intake of macronutrients on the other side showed that the absolute intake of starch was the only independent predictor of DI and ADI. Therefore, starch was the only nutrient included in the multivariable analyses.

The results of the multivariable analyses with all variables in the equation and the stepwise forward analyses are given in Table 2. The main findings were the positive associations between DI and use of metformin and NNS, the positive association between ADI and metformin (the “good” dysbiosis), and the negative associations between ADI and NNS and IBSSS (the “bad” dysbiosis).

**Table 2:** Predictors of Dysbiosis Index and Alternative Dysbiosis Index (linear regression analyses)

Independent predictors	Dependent: DI (all variables)			Dependent: DI (stepwise forward)			Dependent: ADI (all variables)			Dependent: ADI (stepwise forward)		
	B (95% CI)	*p*-value	pc	B (95%CI)	*p*-value	pc	B (95% CI)	*p*-value	pc	B (95%CI)	*p*-value	pc
Gender (female/male)	1.01 (0.17; 1.85)	0.19	0.275				−0.96 (−2.61; 0.70)	0.25	−0.137			
Age (years)	−0.01 (−0.05; 0.02)	0.46	−0.088				0.05 (−0.02; 0.13)	0.15	0.172	0.07 (0.01; 0.13)	*0.033*	0.241
BMI (kg/m2)	−0.00 (−0.08; 0.08)	0.95	−0.007				−0.07 (−0.23; 0.09)	0.38	−0.105			
Smoking (never/ previously /daily)	0.36 (−0.08; 0.80)	0.10	0.193				−0.67 (−1.53; 0.19)	0.12	−0.183			
Coffee (cups/day)	−0.02 (−0.16 to 0.13)	0.79	−0.032				0.02 (−0.26; 0.31)	0.87	0.020			
IBSSS (86)	0.00 (−0.002; 0.004)	0.47	0.086				−0.006 (−0.01; 0.00)	*0.039*	−0.244	−0.01 (−0.01; −0.00)	*0.022*	−0.258
Metformin	0.99 (0.22; 1.75)	*0.012*	0.295	1.11 (0.42; 1.80)	*0.002*	0.339	2.53 (1.04; 4.03)	*0.001*	0.374	2.32 (0.93; 3.71)	*0.001*	0.354
Water (unit ^a^)	0.00 (−0.02; 0.02)	0.98	−0.002				−0.01 (−0.04; 0.03)	0.76	−0.037			
Carb. bev. w/ sugar (unit ^a^)	0.20 (−0,05; 0.45)	0.12	0.186				0.13 (−0.37; 0.63)	0.60	0.062			
Total NNS (unit ^a^)	0.035 (−0.00; 0.07)	0.054	0.228	0.04 (0.01; 0.07)	*0.005*	0.311	−0.08 (−0.15; −0.01)	*0.024*	−0.266	−0.10 (−0.16; −0.04)	*0.001*	−0.362
Starch (g/day)	−0.00 (−0.01; 0.00)	0.36	−0.109				−0.00 (−0.02; 0.01)	0.53	−0.076			

*DI* Dysbiosis Index, *ADI* Alternative Dysbiosis Index, *IBSSS* Irritable Bowel Severity Scoring System, *NNS* Non-Nutritive Sweeteners, *pc* partial correlation, *Carb*. bev: Carbonated beverages. ^a^One unit is 100 ml beverages with NNS or 2 tablets NNS for coffee/tea

Gender and BMI and all variables significantly associated with either DI of ADI in the univariate analyses (age, smoking habits, coffee, IBSSS, Metformin, total NNS, starch) were included as predictors of DI and ADI in the regression analyses. The reasons for exclusion of diabetes and some nutrients are explained in the text. All the variables were included in the first step, and then stepwise forward regression analyses were performed

Italicized *p*-values are statistically significant

### The validation group

Fifty-six women and seven men with a mean age of 38.8 (SD 12.4) years were included in the validation group. The mean ADI and IBSSS scores were −1.68 (SD 2.26) and 287 (SD 79). No one used metformin, and information about NNS was not available. Table 3 gives the associations between the ADI and IBS and IBSSS in the test group and the validation groups with comparisons between the groups. IBS and IBSSS were associated with negative ADI scores. The significant associations between ADI and IBSSS in the two groups were of the same order.

**Table 3:** ADI scores and associations with IBS and IBSSS with comparisons between the groups

Variables	ADI	ADI	Statistics
	Test group	Validation group	*p*-value
	(IBS no/yes: no 63/25)	(IBS yes: 63)	
IBS (no / yes)	−0.41 (2.75) / −1.64 (2.77) (*p* = 0.11)Ϯ	−1.68 (2.26)Ϯ	0.013*
IBSSS	rho = −0.304 (*p* = 0.004)	rho = −0.249 (*p* = 0.049)	0.86 #

*ADI* Alternative Dysbiosis Index, *IBS* Irritable Bowel Syndrome, *IBSSS* Irritable Bowel Severity Score System

*One-Way ANOVA with comparisons between the three groups

Ϯ Post hoc comparisons (Tukey) between the validation group and subjects with and without IBS in the test group were *p* = 1.00 and 0.016 respectively

# Univariate analysis of variance with ADI as the dependent variable and IBSSS and group as independent variables. The *p*-value is the interaction between IBSSS and group and indicates no significant difference between the correlations

## Discussion

Based partly on the same data material and the same dysbiosis test, we have previously published that dysbiosis was prevalent in subjects with morbid obesity and not associated with IBS [18]. The new findings in this study were that dysbiosis measured with the producer’s DI was associated with the use of metformin and NNS, but not with the severity of gastrointestinal symptoms measured as IBSSS. Dysbiosis associated with metformin and NNS have been reported in other studies with more complex, resource demanding, and costly methods [4, 5, 11, 12, 15, 27]. Another new finding was that alternative analyses of the producer’s results allowed separations of types of dysbioses; one type was associated with the use of metformin (“good” dysbiosis) and one with IBS and the use of NNN (“bad” dysbiosis). Today’s lack of knowledge about the clinical significance of dysbiosis measured with this test, and the test’s seemingly inability to differentiate between types of dysbioses nearly eliminates its clinical usability. Hopefully, further research will clarify the clinical consequences of dysbiosis and types of dysbioses measured with this test.

Dysbiosis has been attributed a causal role of obesity in animals. The clinical significance of dysbiosis in humans with obesity and for obesity associated disorders such as insulin resistance, glucose intolerance and type 2 diabetes is less clear [1–3, 28]. The relatively weak associations between obesity and dysbiosis and the large interpersonal variation hamper the interpretation of the results [28]. The variations might indicate different types of dysbiosis, e.g. “good” and “bad”. Theoretically, one type of dysbiosis might have favourable and unfavourable effects referring to different outcomes.

Neither has the clinical significance of dysbiosis associated with the diet and use of drugs been clarified [4–7]. Metformin is a drug of particular interest in subjects with morbid obesity because of the anti-hyperglycemic, insulin sensitising, and weight-reducing effects [8, 9]. The drug’s effect on the faecal microbiota is well established [4, 5, 11, 12].The mechanisms by which metformin exerts its effects have until recently been uncertain [8, 29]. Importantly, intravenous administration has no effect in either non-diabetic subjects or subjects with type 2 diabetes [30, 31]. The glucose tolerance improved in germ-free mice given faeces from metformin-treated mice, indicating that the effect in part depends on alteration of the gut microbiome [11, 12]. The metformin-induced dysbiosis is, therefore, “good” for the effect of metformin.

The favourable and unfavourable effects of NNS on body weight, lifestyle, and metabolism is continuously discussed, and the literature is probably heavily biased [14, 32–35]. The dysbiosis caused by NNS induces glucose intolerance and has been linked to obesity by the obesity-associated metabolic changes [15–17]. Therefore, the NNS associated dysbiosis is probably “bad” for subjects with morbid obesity.

IBS is one among many disorders that has been associated with alterations in the gut microbiota [18–21]. The dysbiosis in subjects with IBS is a “bad” dysbiosis since faecal microbiota transplantation may normalise the microbiota and improve symptoms [36]. In all, there are several types of dysbiosis that might be separated into “good” and “bad”.

The producer’s test response does not differentiate between types of dysbiosis. The ADI based on simple explorative analyses of available results in the producer’s report could easily separate the “good” metformin-type dysbiosis from the “bad” NNS-type dysbiosis. The ADI-score was adjusted so that “good” and “bad” dysbiosis had positive and negative scores respectively. If the results are reproducible, and the dysbiosis test allows construction of other clinically relevant dysbiosis indexes, the potential usefulness of the test increases markedly.

The ADI was not constructed to explore dysbiosis associated with IBS and gastrointestinal complaints. The negative correlations between ADI and IBS and gastrointestinal symptoms were therefore new and interesting findings, which were confirmed with unadjusted and adjusted analyses in the validation group. The findings are in accordance with other reports indicating associations between IBS and dysbiosis [19–21]. The ADI could be a test for detection of “bad” dysbiosis in subjects with IBS and gastrointestinal complaints and replace complex, resource demanding and costly 16S gene sequencing.

Further research, aiming at enlarging the producer’s test response with the specification of the type of dysbiosis related to dietary factors, drugs, disorders and diseases (e.g. metformin- or NNS-like, or “good” or “bad”) is desirable. Specified results might predict response to treatment, e.g. antibiotics and other drugs, probiotics, prebiotics, diet, and faecal microbiota transplant. Treatment aiming at prevention or normalising of a “bad” dysbiosis or induction of a “good” dysbiosis could change the treatment of a range of disorders [37].

### Strengths and limitation

The test group and the validation group were consecutive subjects representative of subjects referred to outpatient clinics for morbid obesity and gastrointestinal complaints respectively. Because the ADI was constructed to detect differences between metformin and NNS, the significant differences between the ADI scores for metformin and NNS were expected. It was nevertheless pleasing that the ADI could be constructed so easily. The most impressive findings were the associations between IBS and IBSSS and the negative ADI score. The ADI was not constructed to find these differences, and they were not detected with the producer’s result report. It was a strength that these findings were confirmed in the validation group, which substantiates that a negative ADI indicates a “bad” NNS- or IBS-like dysbiosis.

The external validity could be questioned since the ADI was based on results from subjects with morbid obesity who might have a high prevalence of dysbiosis also without having gastrointestinal comorbidity and use of metformin and NNS. The exclusion of subjects using antibiotics the last month might have been a too short period.

## Conclusions

A commercially available test for faecal dysbiosis showed a high prevalence of dysbiosis in subjects with morbid obesity, particularly in users of metformin and NNS, but no association with gastrointestinal complaints. An ADI based on explorative analyses of the results from the test could differentiate between the “good” dysbiosis associated with metformin and the “bad” dysbiosis associated with NNS. The “bad” dysbiosis was also associated with gastrointestinal symptom severity. The associations between IBS and gastrointestinal symptom severity were confirmed in an independent validation group, indicating that ADI might be a valid diagnostic tool for the diagnosis of IBS-associated dysbiosis. Rather than merely reporting dysbiosis and degrees of dysbiosis, diagnostic tests for faecal dysbiosis should separate between types of dysbiosis.

## Abbreviations

ADI: Alternative Dysbiosis Index
BMI: Body Mass Index
CI: Confidence interval
DI: Dysbiosis Index
FFQ: Food Frequency Questionnaire
IBS: Irritable Bowel Syndrome
IBSSS: Irritable Bowel Severity Scoring System
NNS: Non-Nutritive Sweeteners
pc: partial correlation
SD: Standard Deviation
